# Dynamic Repertoire of Brain Networks in Mindfulness-Based Cognitive Therapy During Rumination: A Randomized Controlled Trial

**DOI:** 10.1016/j.bpsgos.2026.100753

**Published:** 2026-05-12

**Authors:** Anne Maj van der Velden, Jakub Vohryzek, Paulina Clara Dagnino, Willem Kuyken, Jesus Montero-Marin, Guusje Collin, Morten L. Kringelbach, Henricus G. Ruhe

**Affiliations:** a Department of Clinical Medicine, Aarhus University, Aarhus, Denmark; b Department of Psychiatry, University of Oxford, Oxford, United Kingdom; c Department of Psychiatry, Radboud University Medical Center, Nijmegen, the Netherlands; d Donders Institute for Brain, Cognition and Behaviour, Radboud University, Nijmegen, the Netherlands; e Centre for Eudaimonia and Human Flourishing, Linacre College, University of Oxford, Oxford, United Kingdom; f Centre for Brain and Cognition, Computational Neuroscience Group, Department of Information and Communication Technologies, Universitat Pompeu Fabra, Barcelona, Spain; g International Centre for Flourishing, Universities of Oxford (UK), Aarhus (DK), and Pompeu Fabra (Spain); h Teaching, Research and Innovation Unit, Parc Sanitari Sant Joan de Déu, Sant Boi de Llobregat, Barcelona, Spain; i Center for Biomedical Research in Epidemiology and Public Health, Madrid, Spain; j Contemplative Studies Centre, School of Psychological Sciences, University of Melbourne, Melbourne, Australia; k McGovern Institute for Brain Research, Department of Brain and Cognitive Sciences, Massachusetts Institute of Technology, Cambridge, Massachusetts

**Keywords:** Depression, Dynamic brain connectivity, Mechanisms, Mindfulness, Rumination

## Abstract

**Background:**

Depression is a prevalent and debilitating affective disorder characterized by the dominance and persistence of depressive rumination. Mindfulness-based cognitive therapy (MBCT) is an effective treatment for recurrent depression developed specifically to target rumination and recurrence risk by training metacognitive awareness and adaptive attention, emotion, and self-regulation skills. However, the underlying mechanisms by which mindfulness training impacts maladaptive depressive rumination are not well understood, and a deeper understanding of its effects on the complex brain dynamics during depressive rumination is needed.

**Methods:**

In a randomized controlled functional magnetic resonance imaging (fMRI) study (*N* = 80), we examined dynamic neural changes during resting-state fMRI of an experimentally induced rumination state before and after treatment with MBCT (*n* = 27) for recurrent depression in addition to treatment as usual (TAU) or TAU alone (*n* = 21). More specifically, we characterized the changes during a depressive rumination state as a repertoire of metastable substates, each with an occurrence frequency (fractional occupancy) and stability (lifetimes).

**Results:**

We found that MBCT training compared with TAU altered the fractional occupancy of a salience-somatomotor metastable substate during the depressive rumination state. These dynamic network changes in turn were associated with reduced trait rumination posttreatment and reduced depressive symptoms at the 3-month follow-up.

**Conclusions:**

In a ruminative state, changes in the dynamics of the somatosensory-salience network following mindfulness training was associated with improved clinical outcomes and reduced trait rumination, which may provide insight into candidate brain mechanisms or markers of treatment response to MBCT.

## Plain Language Summary

Mindfulness-based cognitive therapy (MBCT) targets rumination as a key risk factor for depression, but MBCT’s effects on brain dynamics during rumination are not well understood. In this randomized controlled study, adults with recurrent depression received MBCT plus usual care or usual care alone and underwent brain scans during a ruminative mind state before and after treatment. MBCT reduced the likelihood of brain dynamics involving salience and somatomotor networks occurring during the ruminative mind state, and these neural changes predicted reduced rumination and depressive symptoms after treatment. These findings may provide insight into possible brain mechanisms behind the clinical benefits of MBCT.

Depression is a leading cause of disability worldwide (1). One of the most debilitating aspects of depression is the dominance and persistence of depressive rumination (2). Depressive rumination is characterized by a persistent, rigid, and self-related focus on depressed mood and its causes and consequences (3). Rumination has been found to predict increased risk of recurrence in remitted individuals, severity of depressive episodes, and risk of developing treatment-resistant and chronic depression trajectories (2,4,5).

Mindfulness-based cognitive therapy (MBCT) is an effective treatment for recurrent depression and is recommended as a preventive treatment in a number of national health guidelines (6, 7, 8). MBCT can reduce the risk of relapse by approximately 50%, but many individuals will remain vulnerable to experiencing residual depressive symptoms, relapse, or recurrence of a full depressive episode. To optimize outcomes, we need to develop a greater understanding of why, when, and for whom MBCT works (9). MBCT was developed to target depressive rumination and recurrence risk by training metacognitive awareness and adaptive attention, emotion, and self-regulation skills (10). More specifically, participants are taught to recognize, decenter, and disengage from ruminative negative thoughts by redirecting attention to the embodied experience of present-moment sensations and relate to the changes in present-moment experience with a nonjudgmental and accepting attitude (11). The ability to recognize, decenter, and disengage from ruminative negative thoughts is in turn expected to result in reduced depressive symptoms and lower relapse risk (11). However, the underlying mechanisms by which mindfulness training impacts maladaptive depressive rumination are not well understood, and therefore a deeper understanding of how MBCT impacts complex neurocognitive processes during depressive rumination is warranted.

Mindfulness-based interventions and practices have been found to alter brain function in regions and circuits that underlie attention, interoception, emotion regulation, and self-relevant processing [for reviews, see (12, 13, 14, 15)]. Areas in the salience network and default mode network have especially been implicated in mindfulness training, rumination, and depressive symptomatology (2,12,15, 16, 17). However, research on the neural mechanisms of MBCT specifically for recurrent depression and depressive ruminative mind states is still very scarce (18). To our knowledge, in the first neurocognitive study on depressive rumination in relation to MBCT, we found that MBCT compared with treatment as usual (TAU) led to decreased salience network connectivity to the lingual gyrus during a ruminative state and that this change in salience network connectivity mediated improvements in the ability to sustain and control attention to body sensations (18) when using static connectivity measures. While these findings indicate that reduced salience network connectivity may play a role in how likely participants are to get stuck in persistent ruminative processing, static connectivity measures cannot elucidate how stable or variable the connectivity patterns are over time, which we hypothesize to be key to understanding and disentangling the complex and subtle neural dynamics involved during depressive rumination (4).

Recently, new methods have been developed to account for the dynamic nature of whole-brain activity (19,20). While sliding-window approaches have shown the importance of time-varying brain activity, more recent methods have exploited moment-to-moment brain activity with the resolution of the sampling frequency of functional magnetic resonance imaging (fMRI) (21, 22, 23). Here, we focus on the Leading Eigenvector Dynamic Analysis (LEiDA) that uses instantaneous phase relationship between regions to describe a repertoire of metastable substates (phase-locking patterns) evolving in time, thus making it possible to characterize changes in the depressive rumination brain state in terms of dynamic measures such as the probability of occurrence (fractional occupancy) and stability (lifetimes) of the metastable substates (22,24). LEiDA has been successfully applied to many neurological and psychiatric conditions (25, 26, 27, 28, 29, 30), health (24,28,31), and aging (22,30), as well as in studies focusing on changes to brain activity due to pharmacological, electrical, and in silico perturbations (32, 33, 34, 35, 36, 37). Therefore, in our context, a dynamic approach can elucidate the flexibility or rigidity of metastable substates during a ruminative mind state (37). Hence, functional dynamic connectivity methods may yield valuable insight into how neural dynamics during a ruminative state may change on a more subtle level after a mindfulness-based intervention in a way that cannot be captured by traditional static functional connectivity measures looking at an average connectivity (4).

Thus, we aimed to investigate how MBCT impacts neurocognitive dynamics during rumination by applying dynamic functional connectivity in a secondary analysis of a randomized controlled fMRI trial with MBCT+TAU versus TAU for recurrent depression (18). Specifically, we aimed to investigate how mindfulness training impacts the probability of occurrence and duration of the key brain metastable substates present in a ruminative state and whether changes in these neural dynamics are associated with clinical outcomes.

## Methods and Materials

### Study Design and Participants

**Figure 1:**
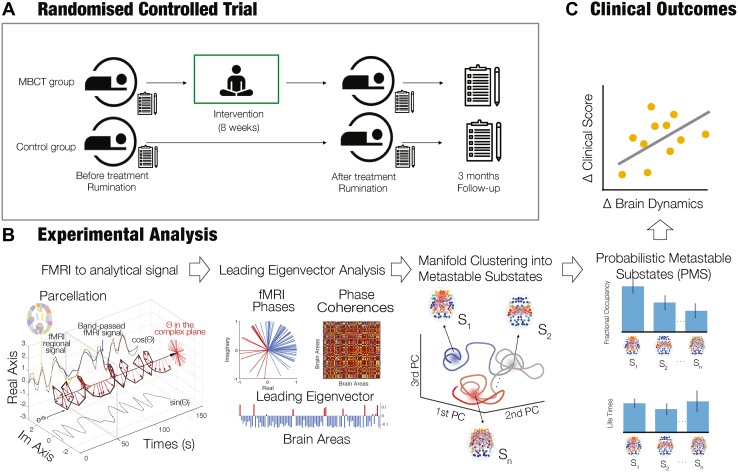
Experimental design and Leading Eigenvector Decomposition Analysis (LEiDA) pipeline. **(A)** Study design including randomization, functional magnetic resonance imaging (fMRI) measures at baseline and posttreatment, and clinical data collection at baseline, posttreatment, and 3-month follow-up. **(B)** The Automated Anatomical Labeling atlas–parcellated fMRI time series were transformed into the Hilbert-transformed signal. Instantaneous phase was extracted for each region at every time point of the recording, and an instantaneous phase coherence matrix was computed and decomposed to obtain the leading eigenvectors for each time point of the recording. Unsupervised clustering was performed to obtain the LEiDA metastable substates, and then dynamic measures of fractional occupancy and lifetimes duration were computed. [Figure adapted with permission from Lord *et al.* (31).] **(C)** Comparing change in fMRI measures (pre- and posttreatment) with changes in depressive symptoms and rumination (pre- and posttreatment and 3-month follow-up). MBCT, mindfulness-based cognitive therapy; PC, principal component.

After approval of the study protocol by the Regional Ethics Council of Central Jutland, Denmark, and obtaining written informed consent from the participants, patients participated in a randomized controlled trial examining change in (neurocognitive) functioning between MBCT+TAU versus TAU only (see Figure 1). The trial has been described in full elsewhere (18) and is registered at ClinicalTrials.gov (NCT03353493).

Eighty adult participants with a diagnosis of recurrent major depressive disorder with or without a current episode were recruited. An independent researcher randomly allocated (5:3 ratio) participants using a computerized system. Randomization was stratified for antidepressant usage and participants’ symptomatic status. The researchers conducting MRI scans were masked to treatment allocation. Questionnaires were administered online.

### Intervention

MBCT+TAU is an 8-week manualized group-based intervention, which combines psychoeducational elements from cognitive behavioral therapy for depression with systematic training in mindfulness meditation (see further outline of the program in Figure S7). The program consists of weekly classes of 120 minutes with daily homework. The program was taught in university settings by highly experienced therapists fulfilling internationally recognized good practice guidelines for teachers, trainers, and supervisors of mindfulness courses (38). The teachers were both supervisors and trainers of other mindfulness teachers and had previously obtained the highest available rating (advanced) on the Mindfulness-based Interventions Teaching Assessment Criteria (MBI-TAC) measure (39). TAU was restricted to no psychotherapeutic intervention and either a stable dose of antidepressant medication or no medication. TAU participants were offered MBCT after they completed all study assessments. In the MBCT+TAU group, we also recorded engagement with the treatment. Mean attendance was above 7 for 8 sessions and above 3 days of practice per week (Table S1).

### Measures and Procedures

Participants were assessed with questionnaires and MRI scans before and after treatment, and depressive symptoms were measured again at the 3-month follow-up.

### Psychological Processes and Clinical Measures

We assessed depressive symptoms using the Quick Inventory of Depressive Symptomatology-Self-Report (QIDS-SR) (40); perceived stress using the Perceived Stress Scale (10); interoceptive awareness using the subscales of noticing, emotional awareness, body listening, attention regulation, trusting, and not distracting of the Multidimensional Assessment of Interoceptive Awareness (41); decentering using the Experiences Questionnaire, decentering factor (42); mindfulness skills using the Five Factor Mindfulness Questionnaire short form (43); and trait rumination using the Rumination Response Scale (RRS) (4).

### fMRI Paradigm and Rumination Induction

The fMRI paradigm during a rumination state was part of a larger paradigm including an initial structural scan followed by 4 separate functional connectivity scans (5 minutes each) in the consecutive order of resting state, an instructed mindfulness state, resting state, and an instructed rumination state. Here, we focus on the changes by treatment (time × group) in the rumination state, as our aim is to explore whether, and how, MBCT+TAU impacts neural dynamics of the probability of occurrence and stability of key metastable substates during a ruminative state.

In the rumination state, participants were guided through a rumination induction adapted from Karl *et al.* (44) in which participants first rehearsed a self-selected sad autobiographical memory and subsequently were instructed to stay with their sad mood and reflect on self-related causes and consequences of their low mood. The rumination induction paradigm has been validated and is known to induce negative self-related thoughts in individuals with a history of recurrent depression (44).

After the rumination state as well as during the prior resting and mindfulness states, participants were asked to rate awareness of negative thoughts (“I had negative thoughts about myself”) and body awareness (“I was aware of my body”) using a visual analog scale in the scanner (0%–100% sliding scale indicating level of agreement). This enabled us to validate and access the rumination paradigm’s ability to induce negative self-related thoughts at each time point and between groups (Figure S1). We also checked that participants were able to follow the instructions.

As requested by the ethical committee, the rumination condition of the fMRI paradigm was voluntary. Before scanning at each time point, we instructed participants about the nature of the task and highlighted its voluntary nature. In addition, there was a brief follow-up interview with a clinically trained member of the research team, the purpose of which was to make sure that participants were well after the experiment.

### MRI Acquisition and fMRI Preprocessing

Structural and functional images of the brain were acquired on a 3T Siemens Magnetom Skyra scanner with a 32-channel head coil (using software version Scout; Siemens Healthineers). For preprocessing, we used FSL tools (version 6.0). Preprocessing steps followed standard procedures, including 1) registering the functional to the structural image, 2) registering the structural image to standard space, 3) motion correction, 4) spatial smoothing, and 5) high-pass filtering (100-second cutoff). For complete details of the preprocessing, see the Supplement.

### Leading Eigenvector Dynamics Analysis

We applied LEiDA to examine dynamic changes in a rumination state in patients undergoing MBCT+TAU versus TAU. For each participant and condition (pre- and postintervention scans in the rumination state), we took each parcellated region’s fMRI signal (Automated Anatomical Labeling atlas of 90 regions), bandpass filtered it, and used the Hilbert transform to obtain the instantaneous amplitude and phase at each time point of the recording. For all participants, the phase coherence matrix between brain areas was computed at each time point, and the dimensionality of each matrix was reduced to its leading eigenvector. K-means clustering was applied to the leading eigenvectors, and the repertoire of metastable substates was obtained (Figure 1B). Then, we determined the probability of occurrence (fractional occupancy) and the duration (lifetimes) of underlying metastable substates before and after treatment for both groups to compute the probabilistic metastable substate (PMS) space for each session. The optimal number of cluster centers was chosen based on 1) optimal clustering performance as determined by a silhouette score (a control measure quantifying the cohesion of the clusters), 2) the functional relevance of PMS as measured by the correlation of each metastable substate with the known 7 resting-state networks, and 3) the lowest clustering solution that would result in a Bonferroni-corrected statistical significance. For a detailed methodological description of LEiDA and the analysis, see the Supplement.

### Comparisons With Psychological Processes and Clinical Outcomes

We compared changes in fractional occupancy and lifetime duration of metastable substates (significantly different between groups over time) with changes in psychological processes and clinical outcomes using Spearman correlations, which are robust to identifying monotonical nonlinear relationships (Figure 1C; for completeness, in Table S6, we report both Pearson and Spearman correlations as well as partial correlations correcting for age, sex, antidepressant usage, and baseline depressive symptoms). Subsequently, we followed up with a regression analysis looking at whether such a relationship was specific to the MBCT+TAU group compared with the TAU group and as such may provide insight into candidate neural mechanisms or markers of treatment response.

## Results

For the study, 80 participants were randomly allocated to receive MBCT+TAU (*n* = 50) or TAU alone (*n* = 30) (Figure 1). We had 66 participants adhering to treatment allocation who completed postscanning sessions including the rumination induction. Of these, 27 MBCT+TAU and 21 TAU participants completed the rumination task (Figure 1). The baseline characteristics were similar in the 2 groups (Table S4). Also, see the CONSORT (Consolidated Standards of Reporting Trials) diagram of participant flow in Figure S6.

Out of ethical concerns, the rumination condition of the fMRI paradigm was voluntary. The participants who did not participate in the rumination condition (27% from the total sample completing both scans) had similar baseline characteristics except for higher depressive symptoms and age (Table S4). The clinical range in both cases covered asymptomatic to severe depression, but among those who did not participate in the rumination condition, there were more participants who were severely depressed, and fewer participants were below the asymptomatic threshold (Table S4 and Figure S2A). After MBCT+TAU, those who declined the rumination task and those who participated in the rumination task experienced a reduction in depressive symptoms and had comparable lower clinical scores posttreatment (Figure S2B). For both groups, the rumination induction paradigm was effective in inducing negative self-related thoughts (Figure S1).

### Analyzing Changes in Fractional Occupancy and Lifetime Duration During Rumination

We chose *k* = 8 by examining the optimal clustering performance of the PMS as determined by the lowest clustering solution that resulted in Bonferroni-corrected statistical significance for the fractional occupancy measure (Figure S3A, B) and the silhouette score (Figure S3C).

**Figure 2:**
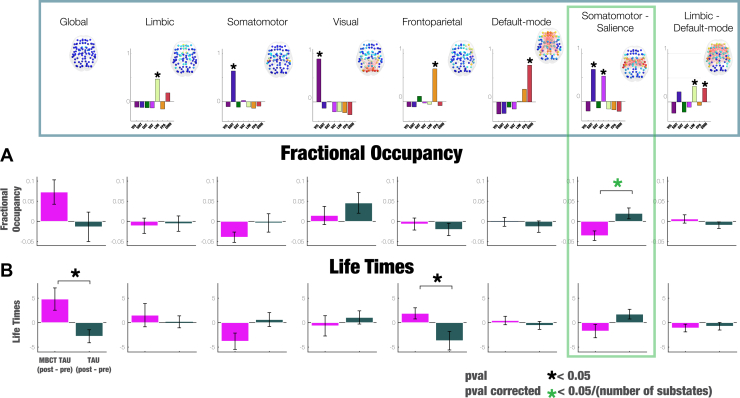
Repertoire of recurrent metastable substate in neuroimaging data of mindfulness-based cognitive therapy (MBCT) + treatment as usual (TAU) and TAU groups. **(A)** Chosen clustering solution of 8 metastable substates. We found a significant decrease in fractional occupancy of a salience-somatomotor metastable substate (*p* = .0055, corrected for multiple comparisons across 8 metastable substates [*p* = .044]) comparing MBCT+TAU post-pre vs. TAU post-pre. Green asterisk signifies significant Bonferroni-corrected *p* values (<.05/8). **(B)** We found a trend of an increase in lifetime duration of the global metastable substate and the frontoparietal metastable substate (*p* = .012 and *p* = .019, respectively, uncorrected for multiple comparisons) comparing MBCT+TAU post-pre vs. TAU post-pre. Assessment of the difference between groups was performed with independent samples *t* tests, in this specific clustering solution (*k* = 8) for fractional occupancy and lifetime duration. Black asterisk signifies significant uncorrected *p* values (<.05).

**Figure 3:**
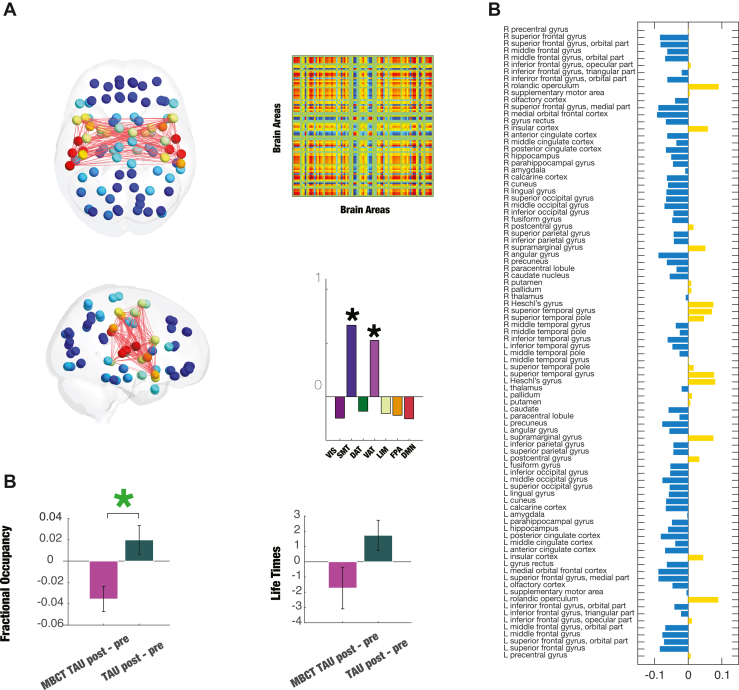
Change in the fractional occupancy of the salience-somatomotor metastable substate. **(A)** Cortical renderings of the salience-somatomotor metastable substate (seventh metastable substate in the *k* = 8 clustering solution). The matrix represents coherence between Automated Anatomical Labeling atlas nodes and their rendering on the resting-state networks as defined by Thomas Yeo *et al.* (58). Black asterisk signifies significant uncorrected *p* values (<.05). **(B)** Cortical labels for the salience-somatomotor metastable substate (in yellow are the regions associated with this metastable substate). **(C)** Decrease in fractional occupancy of the salience-somatomotor metastable substate after mindfulness-based cognitive therapy (MBCT) + treatment as usual (TAU) vs. an increase in the TAU group. No differences between groups were found for the lifetime duration of this metastable substate. Green asterisk signifies significant Bonferroni-corrected *p* values (<.05/8). DAT, dorsal attention network; DMN, default mode network; FPA, frontoparietal network A; L, left; LIM, limbic network; R, right; SMT, somatomotor network; VAT, ventral attention network; VIS, visual network.

Then, we analyzed the fractional occupancy and lifetime duration for each of the metastable substates. The *p* values resulting from between-group comparisons (using the independent samples *t* test) of the post- minus pretreatment fractional occupancies and lifetimes (i.e., change over time) per group are shown in Figures 2 and 3 and Figure S8B. For the chosen clustering solution of 8 PMSs, a significant decrease in the fractional occupancy of the salience-somatomotor metastable substate (*p* = .044) was observed after correcting for multiple comparisons for MBCT+TAU versus TAU (Figure 2A). This difference in the fractional occupancy of this metastable substate was consistent over a range of clustering solutions *k* = 7 to *k* = 20 (except *k* = 11 and *k* = 17), with *k* = 8 and *k* = 9 showing the most robust differences for the Bonferroni-corrected *p* values (Figure S3A, B). This salience-somatomotor metastable substate consisted of not only bilateral areas of the salience network (i.e., the insula) and several areas of the somatosensory and motor networks (i.e., Rolandic operculum, supramarginal gyrus, frontal inferior operculum, temporal superior gyrus, precentral and postcentral gyrus, putamen) but also auditory areas (i.e., Heschl’s gyrus, areas of the temporal pole [superior temporal gyrus]) and part of the basal ganglia such as the pallidum (Figure 3).

**Figure 4:**
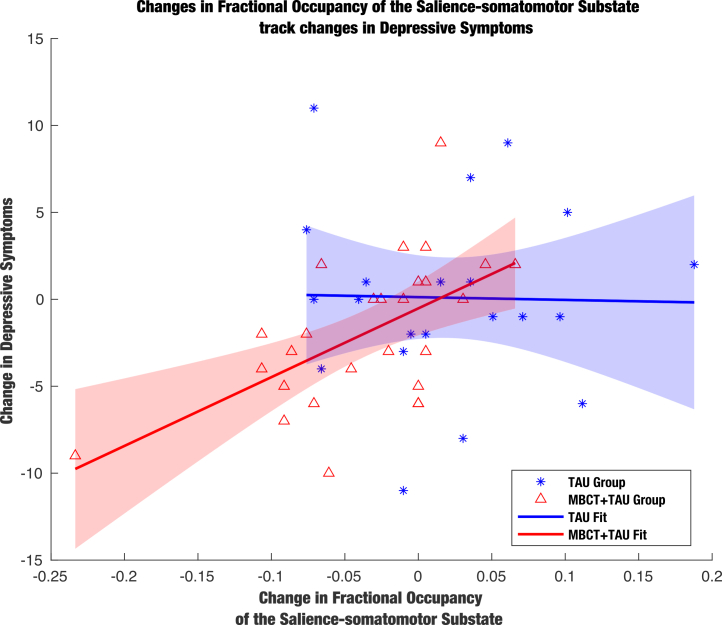
Changes in the fractional occupancy of the salience-somatomotor metastable substate post minus pretreatment are associated with changes in depressive symptoms at the 3-month follow-up. Change in the fractional occupancy of the salience-somatomotor metastable substate (posttreatment − pretreatment) in the mindfulness-based cognitive therapy (MBCT) + treatment as usual (TAU) group was significantly associated with changes in depressive symptoms (Quick Inventory of Depressive Symptomatology, 3-month follow-up − pretreatment), while this was not the true for the TAU group. The figure shows the linear fit of both the MBCT+TAU and TAU groups with the 95% CIs. Only the association between reduced fractional occupancy of the salience-somatomotor metastable substate and reduced depressive symptoms in the MBCT+TAU group posttreatment and at the 3-month follow-up was specific to the MBCT+TAU group. In Figure S5A, we report the figure without the outlier; in Figure S5B, we report results for trait rumination with and without removing the outlier.

MBCT+TAU attendance and practice during the MBCT treatment were also correlated with change in fractional occupancy (Table S6). Regression analysis showed that treatment was a significant predictor of change in fractional occupancy (β = 0.055, *t*_46_ = 2.917, *p* = .005) also when controlling for covariates of age, sex, antidepressant medication, and baseline depressive symptoms (β = 0.065, *t*_40_ = 3.366, *p* = .002) and when controlling for likelihood of participating in the rumination task (β = 0.064, *t*_40_ = 3.174, *p* = .003). The latter sensitivity analysis used inverse probability-weighted linear regression (45) with weights reflecting participant’s likelihood of completing the rumination task based on baseline QIDS-SR scores and controlled by the same covariates.

For lifetimes, a trend (significant at *p* < .05 before correction for multiple comparisons but not after correction) (Figure 2B) toward increased duration of the global metastable substate and the frontoparietal metastable substate was observed.

### Relating Change in Fractional Occupancy and Lifetimes During Rumination to Psychological Processes and Clinical Outcomes

To understand how the reduction in fractional occupancy of the salience-somatomotor metastable substate in the MBCT+TAU group was related to changes in psychological processes and clinical outcomes, we correlated changes in the salience-somatomotor metastable substate with changes in questionnaire scores. We found associations with reduced depressive symptoms at the 3-month follow-up (ρ = 0.554, *p* = .003) (Figure 4) and reduced trait rumination (RRS total, ρ = 0.410, *p* = .037) (Figure S5), as well as subscales of brooding (ρ = 0.419, *p* = .033) and depression (ρ = 0.420, *p* = .033). These findings were also significant when correcting for age, sex, antidepressant medication, and baseline depressive symptoms and for reduced depressive symptoms (but not rumination) when omitting outliers (Figure S5). Moreover, increased treatment engagement, i.e., attendance (ρ = −0.442, *p* = .021) and practice (ρ = −0.407, *p* = .035), was also related to the reduction in fractional occupancy of the salience-somatomotor metastable substate and for attendance (but not practice) when removing the outlier in fractional occupancy.

To explore differential effects in the MBCT+TAU and TAU groups, we tested this in interaction models. For depressive symptoms, the association with fractional occupancy of the salience-somatomotor metastable substate was significantly different between the MBCT+TAU and TAU groups at 3 months (β = −48.919, *t* = −2.500, *p* = .017) also when adding covariates of age, sex, antidepressant medication, and baseline depressive symptoms to the model (β = −43.410, *t* = −2.364, *p* = .024).

In contrast to the fractional occupancy findings, the trends for lifetime change (i.e., significant before correcting for multiplicity only) did not survive correction for outliers and age, sex, baseline depressive symptoms, and antidepressant medication.

## Discussion

In a randomized controlled trial comparing the effect of MBCT+TAU versus TAU only in individuals with recurrent depression, we examined the dynamic changes during a rumination state. We found that MBCT+TAU altered the probability of occurrence (fractional occupancy) of a metastable substate composed of areas of the salience and somatosensory network. These dynamic changes in turn were associated with reduced trait rumination after treatment and decreased depression symptoms at the 3-month follow-up and were robust to corrections for age, sex, baseline depressive symptoms, antidepressant medication, outliers, and likelihood of participating in the rumination task. While we also found a trend in the MBCT+TAU group altering the duration (lifetimes) of metastable substates, this pattern was not robust as it did not survive correction for multiple comparisons.

The findings are consistent with a growing body of literature implicating the salience network in depressive symptomatology and treatment response (16,17,46,47). Changes in areas of the salience network such as the insula have been linked to prediction of treatment response (48,49) across several forms of psychotherapy and pharmacotherapy for depression, as well as treatment response to mindfulness-based interventions across a wide group of populations (50,51). In addition, it has been suggested that the salience network plays a key role in valence, affective biases, and persistence of depressive rumination (2). We also found areas of the somatosensory and motor networks (i.e., Rolandic operculum, supramarginal gyrus, frontal inferior operculum, temporal superior gyrus, central gyrus, pallidum) and subcortical areas (e.g., putamen in the basal ganglia) that together have been implicated in reward and motivational circuits in depression, emotional processing, and severity of depressive symptoms and recurrence risk (52, 53, 54, 55).

In this secondary analysis, we used a model-free, bottom-up, and data-driven approach to identify MBCT-specific changes in the repertoire of metastable substates during rumination. In contrast to the previous static analysis (18) that measured average change in functional connectivity after MBCT, this choice of methodology enabled us to detect the more subtle metastable substate changes during rumination that were associated with reductions in depressive symptoms and trait rumination. Furthermore, a similar salience-somatomotor metastable substate difference was found with LEiDA in expert meditators during meditation (56), and we observed an indication of an inverse dose-response relationship with attendance and practice of MBCT. Therefore, salience-somatomotor metastable substate changes may be implicated in potential meditation practice effects.

While the design of our rumination task does not allow us to draw clear conclusions about the underlying cognitive changes without engaging in reverse inference, we speculate that the reduced probability of occurrence of the salience-somatomotor metastable substate may play a role in reducing the persistence and stickiness of the ruminative mind state. On a neural level, it has been proposed that particular regions of the salience mode network may be related to persistence, stickiness, and inability to disengage from the ruminative state persistence (2), and some theories suggest that changes in sensory processing and somatomotor regions may also play a part in a persistent rumination (11). Furthermore, it has been suggested that subcortical areas such as the putamen, which was involved in this salience-somatomotor metastable substate, may play a role in habitual negative thinking patterns in depression (54,55). Theoretically, MBCT targets the ability to recognize, decenter, and disengage from ruminative thoughts by redirecting attention to present-moment body sensations rather than aiming to change ruminative thought content (11). Likewise, in this study, we saw that the prevalence of negative thoughts during rumination remained high after treatment and across groups despite clinical improvement in the MBCT group. Therefore, it may not be the prevalence of negative self-related thought content that changed during the rumination state as much as it was the persistence and stickiness of the rumination state, which in turn may play a role in improved clinical outcomes.

The current study has several strengths and limitations. First, our choice of TAU as the control group is both a strength (generalizability, external validity) and a limitation (lack of specificity in the control condition). However, given the absence of a specific active control group, we cannot know whether the treatment effects are specific to MBCT+TAU treatment or whether other treatments may yield similar dynamic effects. Future research could address this by comparing MBCT with equally effective treatments and examine the extent to which the mindfulness meditation components of MBCT drive the dynamic connectivity change by using either a dismantling design or active attention control.

Second, we used highly experienced MBCT teachers fulfilling internationally recognized good practices guidelines for teachers, trainers, and supervisors of mindfulness courses (38). However, we did not directly measure their adherence to the treatment protocol and teaching competency as teachers were both supervisors and trainers of other mindfulness teachers and had previously been awarded the advanced, highest-available rating on the MBI-TAC measure (39).

Third, out of ethical concerns, participants could choose to opt out of the rumination condition and participate in the rest of the study. While this may introduce selection bias, we found that those choosing not to participate in the rumination condition had higher age and depressive symptoms at baseline, but did not differ on other measures, and the clinical range in both cases covered asymptomatic/mild to severe depression. After MBCT treatment, both those who declined the rumination task and those who participated in the rumination task experienced a reduction in depressive symptoms and had comparable lower clinical scores posttreatment, and the results remained robust when we conducted sensitivity analyses controlling for age, sex, baseline depressive symptoms, and antidepressant medication and using inverse probability weighting adjustment of the regression models based on likelihood to participate in the rumination task.

Given the novelty of the design, we based our statistical power estimation on the COBIDAS (Committee on Best Practice in Data Analysis and Sharing) guidelines on transparent and reproducible neuroimaging research, which used the Neurosynth database to estimate average fMRI power, suggesting 30 participants per group for a medium to large effect. However, this estimation did not consider the power needed for the other measures, and as the fMRI paradigms are based on contrasting across different tasks, states, or groups, such generic power estimations may not be fully applicable when comparing the same individuals entering the same states before and after treatment, with the only difference being the treatment allocation. Therefore, it is possible that the study may be underpowered to find smaller effects.

Another limitation is that each participant was scanned for only 5 minutes during the rumination condition before and after treatment. Longer acquisitions would likely afford a more comprehensive sampling of the recurrent configurations of whole-brain dynamics (57).

### Conclusions

MBCT+TAU compared with TAU led to decreased probability of occurrence of a metastable substate consisting of areas of the salience-somatomotor network and subcortical regions during a ruminative state, and this dynamic change was associated with reduced trait rumination posttreatment and reduced depressive symptoms at the 3-month follow-up and therefore may play a mechanistic role in improving treatment outcomes.

## Supplementary Material

Supplemental Methods, Figures S1–S6, and Tables S1–S7

Key Resources Table
