# Supplementary material for: Dynamic Repertoire of Brain Networks in Mindfulness-Based Cognitive Therapy During Rumination: A Randomized Controlled Trial
Source: Biol Psychiatry Glob Open Sci. 2026 May 12;6(5):100753. doi: 10.1016/j.bpsgos.2026.100753 (PMC13382308; doi:10.1016/j.bpsgos.2026.100753)
Supplement: Supplemental Methods, Figures S1–S6, and Tables S1–S7 [file mmc1.pdf]

SUPPLEMENTARY INFORMATION

Dynamical Repertoire of Brain Networks in Mindfulness-Based Cognitive Therapy During Rumination: A Randomized Controlled Trial

van der Velden *et al.*

S1: Extended Methods and Materials

Methods and Materials

Study design and participants

After approval by the Regional Ethics Council of Central Jutland, Denmark and written informed consent, patients participated in a randomised controlled trial examining change in (neurocognitive) functioning between Mindfulness-Based Cognitive Therapy in addition to treatment as usual (MBCT+TAU) versus TAU only (see Figure 1). The trial has been described in full elsewhere (18) and registered at ClinicalTrials.gov (NCT03353493).

Eighty adult participants with a diagnosis of recurrent major depressive disorder with or without a current episode were recruited. An independent researcher randomly allocated (5:3 ratio) participants using a computerised system. Randomization was stratified for antidepressant usage and participants' symptomatic status. The researchers conducting MRI scans were masked to treatment allocation. Questionnaires were administered online.

Intervention

MBCT+TAU is an 8-week manualized group-based intervention, which combines psychoeducational elements from cognitive behavioural therapy for depression with a systematic training in mindfulness meditation (see further outline of the programme in Supplementary Section Figure S7). The programme consists of weekly classes of 120 minutes with daily homework. The programme was taught in university settings by highly experienced therapists fulfilling internationally recognized 'good practice' guidelines for teachers, trainers and supervisors of mindfulness courses (1). The teachers were both supervisors and trainers of other mindfulness teachers and had previously obtained the highest available rating (advanced) on the Mindfulness-based Interventions Teaching Assessment Criteria Measure (MBI-TAC, (2)). TAU was restricted to no psychotherapeutic intervention and either a stable dose of antidepressant medication or no medication. More specifically, TAU refer to treatment as usual following Danish National Clinical Guideline at time of recruitment, but within the TAU spectrum there is variation in whether or not participants received antidepressant medication (84%) and if they did which kind and doses (majority received SSRIs and SNRIs which are the first treatment recommended in these guidelines). In the clinical guidelines all individuals with a history of recurrent depression will be offered preventative or maintenance antidepressant medication starting with SSRIs then SNRIs, but some will decline or withdraw from medication due to lack of effects or side effects, and therefore the exact combination of medication will vary. However, key to the study design, was that the

participants on antidepressant medication were to be on a dose of medication and not to change treatment in the study period, so the TAU components would not change before and after treatment. TAU-participants were offered MBCT after they completed all study assessments. In the MBCT+TAU group, we also recorded engagement with the treatment. Mean attendance was above 7 for 8 sessions and above 3 days of practice per week (Supplementary Table S1).

Measures and procedures

Participants were assessed with questionnaires and MRI-scans before and after treatment and depressive symptoms were measured again at three months follow up.

Psychological processes and clinical measures

We assessed depressive symptoms using the Quick Inventory of Depressive Symptomatology (QIDS_SR); perceived stress using the Perceived Stress Scale (PSS); interoceptive awareness using the subscales of noticing (N0), emotional awareness (EA), body listening (BL), attention regulation (AR), trusting (TR) and not-distracting (ND) of the Multidimensional Assessment of Interoceptive Awareness (MAIA); decentering using the Experiences Questionnaire (EQ) – decentering factor; mindfulness skills using the Five Factor Mindfulness Questionnaire short form (FFMQ-15), and trait rumination using the Rumination Response Scale (RRS).

fMRI paradigm and rumination induction

The fMRI paradigm during a rumination-state, was part of a larger paradigm including an initial structural scan followed by four separate functional connectivity scans (5 minutes each) in the consecutive order of resting state, an instructed mindfulness-state, resting state, and an instructed rumination-state. Here, we focus on the changes by treatment (time*group) in the rumination state, as our aim is to explore whether, and if so how, MBCT+TAU impacts neural dynamics of probability of occurrence and duration of key metastable substates during a ruminative state.

In the rumination state, participants were guided through a rumination induction adapted from Karl et al (3) in which participants first rehearsed a self-selected sad autobiographical memory and subsequently were instructed to stay with their sad mood and reflect on self-related causes and consequences of their low mood. The rumination induction paradigm has been validated and is known to induce negative self-related thoughts in individuals with a history of recurrent depression (3).

After the rumination state as well as during the prior resting and mindfulness states, participants were asked to rate awareness of negative thoughts ('I had negative thoughts about myself') and

body awareness using a visual analogue scale in the scanner (0-100 percent sliding scale indicating level of agreement). This enabled us to validate and access the rumination paradigm's ability to induce negative self-related thoughts at each time point and between groups (Supplementary Figure S1).

As requested by the ethical committee, the rumination condition of the fMRI paradigm was voluntary. Before undergoing scanning at each timepoint, we instructed participants about the nature of the task and highlighted its voluntary nature. All participants were offered to complete all conditions or to choose not to do the last condition (rumination) before each scanning session. In addition, there was a brief follow-up interview with a clinically trained member of the research team; the purpose of which was to make sure participants were well after the experiment.

MRI acquisition

Functional and structural images of the brain were acquired on a 3 Tesla Siemens Magneton Skyra 3T scanner (Siemens, Erlangen, Germany, software version Scout) using a 32-channel head coil, and using conventional acquisition parameters as specified below.

Structural MRI: A structural three-dimensional T1-weighted (3D-T1) scan was acquired with the following parameters: 176 slices covering the whole brain, TE (echo time)/TR(repetition time) = 3.8/2300 ms, inversion time = 31260 ms, flip angle = 8°, Field of View (FOV) = 256 mm, spatial resolution 1 × 1 × 1 mm3, Generalised Autocalibrating Partially Parallel Acquisitions (Grappa) = 2, and phase-encoding direction = AP.

Functional MRI: The duration of the rumination state was five minutes with 203 volumes of 2D gradient-echo EPI fMRI data were acquired with the following parameters: 52 ascending axial slices covering the whole brain, 3.8 × 3.8 × 3.8 mm3, FOV 192, Grappa = 2, Multiband = 2, TE/TR = 30/1480 ms, flip angle = 65°, and phase-encoding direction = AP.

FMRI preprocessing

We used FSL tools (https://fsl.fmrib.ox.ac.uk/fsl/docs/#/) for preprocessing. Preprocessing steps followed standard procedures and included: skull-stripping (BET tool), registering the functional to the structural image (FLIRT tool with default settings for Boundary-Based registration), registering the structural image to standard space (FNIRT tool with default settings for 12 degrees of freedom and warp-resolution of 10mm), motion correction (MCFLIRT tool) and spatial smoothing of the data with a 5mm kernel. We used an independent component analysis (ICA)-based strategy for Automatic Removal of Motion Artefacts (ICA-AROMA). For further denoising, the first five eigenvariates of time courses extracted from white matter and cerebrospinal fluid

masks (segmentation was done using FAST tool) were removed (using fsl_glm). Finally, data was high-pass filtered (100 seconds cut-off).

Leading Eigenvector Decomposition Analysis (LEIDA)

In this study, we applied Leading Eigenvector Decomposition Analysis (LEiDA) in order to elucidate dynamic changes in large-scale brain networks in patients undergoing MBCT treatment in ruminative brain states, and to describe the properties of the metastable substates, namely Fractional Occupancy (the percentage of frames assigned to a state for the whole of the rumination scan) and Life Times (the mean duration of temporally continuous runs of state occupancy). Importantly, the Probabilistic Metastable Substate (PMS) space - metastable substates with their associated probabilities - were derived specifically for the rumination condition, ensuring a "common-space" in which all the different subjects, conditions and spatially defined metastable substates can be compared."

LEiDA is based on the phase relationship between brain regions at a given time. We used the fMRI signal to calculate the instantaneous phases and amplitudes for each brain region. In detail this meant that for each participant and condition separately (pre- and post-intervention scans in the ruminative state), we took each region's signal based on the AAL parcellation (90 brain regions) and used the Hilbert transform to construct a companion signal that was phase-shifted by 90 degrees. By doing so, we were able to express the signal in its complex form with the angle representing the instantaneous phase information and the magnitude representing its instantaneous amplitude. This analytical approach and the Hilbert transformation are widely used in neuroscience to analyse moment-to-moment changes in oscillatory activity (4).

More specifically, we first obtained the analytical signal for each region n where n = 1… 90 for the length of its time series t. This was calculated by first band-passing the regional signal in a narrow-band of 0.01-0.1 Hz and applying the Hilbert transformation. The analytical signal represents the signal in terms of its instantaneous amplitude and phase, Θ. We use the phase information at every timepoint of the recording to obtain the instantaneous phase coherence (iPC) matrix as follows: $iPC(n,m,t) = \cos(\Theta(n,t)-\Theta(m,t))$. This relationship accounts for the level of phase alignment between individual brain regions. If $\Theta(n,t)-\Theta(m,t) = 0$ then $\cos(0) = 1$ and the regions are fully phase aligned, if $\Theta(n,t)-\Theta(m,t) = \pi$ then $\cos(\pi) = -1$ and the regions are fully phase anti-aligned, and if $\Theta(n,t)-\Theta(m,t) = \pi/2$ then $\cos(\pi/2) = 0$ and the brain regions are phase orthogonal to each other.

We chose the Automated Anatomical Labelling (AAL) atlas for two reasons. For consistency with previous literature ensuring interpretability of the clusters and results (5-7) as well as for the fact

that the AAL includes the subcortical regions. However, other studies have used different parcellations such as the Oxford-Harvard cortical atlas (8-10) and Power 264 atlas with subcortical regions (11) which also report consistent clusters and associations to the functional networks.

Leading Eigenvector Dynamics

We subsequently computed the leading eigenvectors of each phase-locking pattern (a pattern consisting of regions with similar phase) at every recorded timepoint. To obtain the leading eigenvector dynamics, we applied principal component decomposition (PCA) to each iPC and selected the eigenvector with the strongest contribution thus obtaining leading eigenvectors at every timepoint $V_1(t)$ of dimensions 1xN. It has been shown that the leading eigenvector represents at least 50% of the iPC at each timepoint. Furthermore, each leading eigenvector represents a spatial distribution across the parcellation atlas and can be mapped onto the cortical rendering for visualisation.

Clustering into cortical substates

In order to represent the leading eigenvectors in terms of recurring substates of brain activity in time, we cluster them using the unsupervised learning k-means algorithm. To do so, we concatenated all the timeseries across subjects and conditions to achieve a common space where N was the number of dimensions and (TxSbjxCnd) was the number of datapoints where T, Sbj, Cnd reflects timepoints, Subjects and Condition respectively. We used the cosine distance as a measure of similarity and ran the algorithm for a varying number of cluster solutions - from 2-20 clusters with 100 repetitions. Then, every leading eigenvector, representing specific timepoints, was assigned to a given cluster centroid. Lastly, each centroid in a given clustering solution was associated to a given Yeo et al. (2011) (12) functional network by computing the spatial correlation between the functional networks and the centroids themselves. We defined the number of clusters based on i) optimal clustering performance as determined by a silhouette score (a control measure quantifying the cohesion of the clusters), ii) in terms of the functional relevance of metastable substates as measured by the correlation to the known seven resting-state networks and finally, iii) the lowest clustering solution that would result in a Bonferroni corrected statistical significance.

To thoroughly investigate different partitions, we gradually varied k from 2 to 20. This approach allowed us to analyse PMS that exhibited significant differences in brain activity between the MBCT+TAU intervention and TAU control groups. The number of clusters, k, in the k-means

clustering algorithm determines the range of PMS in the repertoire. By increasing k, more intricate, infrequent, and asymmetric PMS are revealed.

Our clustering approach and the associated measures are derived independently of statistical testing. The clustering is performed across a range of solutions, and only subsequently are the resulting states compared between groups to assess statistically significant differences. In this sense, the approach differs from procedures in which statistical criteria directly inform or define the features being tested. To ensure the results were robust, we checked that the identified state was consistent across a range of clustering solutions, and would survive correction for multiple comparisons, and would be supported not only by statistical significance but also by functional interpretability (i.e., correspondence to resting-state networks) and clustering quality (using in the silhouette scores and the Dunn and Davies-Bouldin indices (Supplementary Figure S3c). Moreover, we checked that the findings could be replicated using permutation testing, indicating consistency across parametric and non-parametric approaches and thereby reducing the likelihood that the results reflect distortions introduced by the clustering or LEiDA pipeline.

Dynamical Measures of Fractional Occupancy and Lifetimes

Fractional Occupancy was calculated as a probability of occurrence of a given substate α (centroids) across the time recording. Formally, fractional occupancy was calculated as follows

$$\Pi_\alpha = \frac{1}{T}\sum_{t=1}^{T} \chi[\bar{x}(t) \in R^\alpha]$$

with χ being the indicator function taken on 1 if the state is on at time t and 0 if this is not the case. Fractional occupancy was calculated for every subject and condition.

Furthermore, we computed Lifetimes that quantifies the amount of time a given subset α is consecutively active

$$LT_\alpha = \frac{1}{p_\alpha}\sum_{1}^{p_\alpha} C_{p_\alpha}$$

where p_α is the number of consecutive periods and C_{p_α} their duration).

Category	Whole-group					Participating in rumination task					Avoiding rumination task				
	value	min	max	std	N	value	min	max	std	N	value	min	max	std	N
**Treatment characteristics**															
**Attendance**	7.103	5	8	0.912	39	7.074	5	8	0.997	27	7.167	6	8	0.718	12
**Practice**	3.769	0	6	2.158	39	3.222	0	6	2.082	27	5.000	1	6	1.859	12

Table S1 - ***MBCT Treatment engagement characteristics.*** *Mean attendance (number of sessions attended out of 8 sessions) and practice (average practice days per week).*

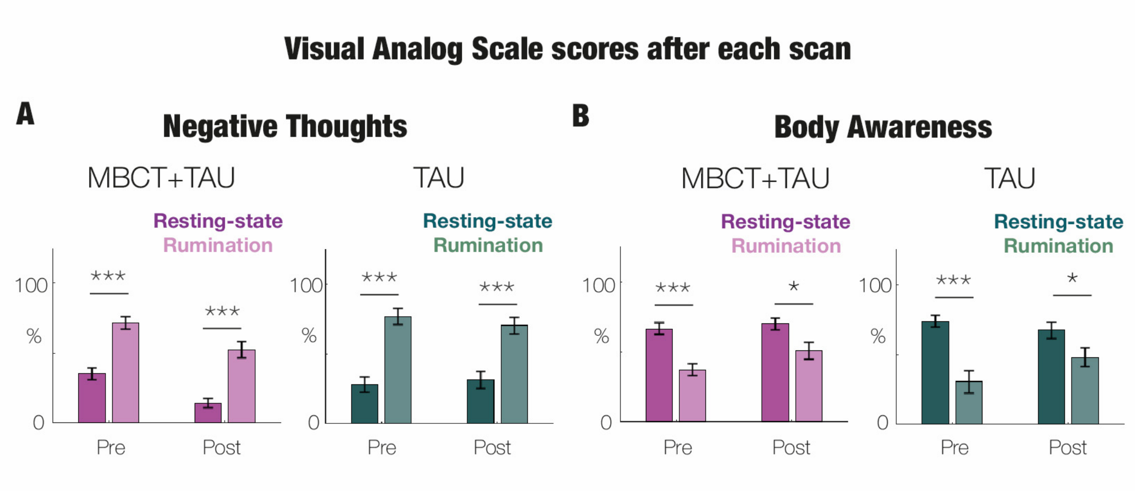

Figure S1 - Visual Analog Scales scores comparing a resting state and the rumination state.
A. *Negative self-related thoughts per time and treatment condition.* ***B.*** *Body awareness per time and treatment condition. Compared to resting state, after the rumination scan, participants reported significantly less body awareness and significantly more negative thoughts about themselves compared to resting-state for all groups (MBCT+TAU or TAU) and sessions (before and after). Reports were made based on visual analogue scale statements e.g. 'I experienced negative thoughts about myself' or 'I was aware of my body' (0-100 percent agreement response). All barplots and error bars show the mean and standard error of the mean, and statistical tests are performed with Wilcoxon rank-sum method. Asterisks represent significance level (*, p-val<0.05; **, p-val<0.01; ***, p-val<0.001). The number of subjects is: resting-state MBCT+TAU N=40, resting-state TAU N=26, rumination MBCT+TAU N=27 and rumination TAU N=21. The number of subjects with no response is 1 for rumination TAU*

Category	MBCT+TAU (N = 50)	TAU (N = 30)
**Sociodemographic Characteristics**	**n = 48**	**n = 28**
**Age, Years**	43.17 (14.22)	45.25 (12.01)
**Sex, Female/Male**	35/15 (70%)	23/5 (82%)
**Educational Level**	**n = 48**	**n = 28**
**Low (<2-year further education)**	15 (30%)	3 (11%)
**Medium (2–4-year further education)**	24 (48%)	21 (75%)
**High (>5-year further education)**	9 (18%)	4 (14%)
**Marital Status**	**n = 48**	**n = 28**
**Married/cohabiting**	43 (90%)	21 (75%)
**Single/Not cohabiting**	5 (10%)	7 (25%)
**Occupational Status**	**n = 48**	**n = 28**
**Employed**	24 (50%)	14 (50%)
**Unemployed/benefits**	10 (20%)	4 (14%)
**Student**	3 (6%)	1 (4%)
**Retired**	7 (15%)	4 (14%)
**Other**	9 (19%)	5 (18%)
**Clinical Characteristics**	**n = 48**	**n = 28**
**Symptomatic (QIDS > 5)**	43 (83%), n = 50	25 (76%), n = 28
**Antidepressant Usage**	43/7 (86%), n = 50	21/7 (75%), n = 28
**Childhood Trauma**	58.79 (6.22), n = 42	58.96 (6.33), n = 26
**Previous Episodes of Depression**	3.90 (1.44), n = 41	3.80 (1.36), n = 23
**Outcomes**	**n = 48**	**n = 27**
**QIDS**	9.23 (4.58)	9.68 (5.10)
**EQ**	31.43 (7.12)	31.26 (7.06)
**MAIA_AR**	17.22 (5.03)	17.78 (4.99)
**MAIA_BL**	6.25 (2.07)	7.40 (3.25)
**MAIA_TR**	8.89 (3.31)	8.40 (3.77)
**MAIA_NO**	12.79 (2.61)	13.96 (3.38)
**MAIA_ND**	9.17 (2.64)	9.01 (2.45)
**MAIA_EA**	15.32 (3.51)	16.57 (4.23)
**FFMQ**	44.21 (8.88)	45.33 (8.02)
**RRS**	53.38 (9.80)	57.51 (8.24)

Table S2. Baseline characteristics per group. *Abbreviations: AR, attention regulation; BL, body listening; EA, emotional awareness; EQ, Experience Questionnaire; FFMQ, Five Factor Mindfulness Questionnaire; MAIA, Multidimensional Assessment of Interoceptive Awareness; ND, not-distracting; NO, noticing; QIDS, Quick Inventory of Depressive Symptomatology.*

Category	Participating in rumination task					Avoiding rumination task				
	value	min	max	std	N	value	min	max	std	N
**Sociodemographic characteristics**										
**Age**	**41.521****	18	66	12.788	48	**51.667****	37	70	9.798	18
**Sex (M/F)**	10/38	-	-	-	48	3/15	-	-	-	18
**Clinical characteristics**										
**Symptomatic (QIDS > 5)**	76.087	-	-	-	46	94.444	-	-	-	18
**mADM**	81.250	-	-	-	48	94.444	-	-	-	18
**Childhood trauma**	39.116	25	90	14.136	43	39.500	25	97	18.608	16
**Outcomes**										
**QIDS**	**8.370****	0	18	4.368	46	**12.556****	5	21	4.655	18
**FFMQ**	44.340	27	61	7.755	47	42.941	32	63	8.678	17
**PSS**	20.277	9	31	6.258	47	23.588	11	34	5.938	17
**RRS**	55.596	29	73	9.786	47	52.771	32	68	9.750	17
**EQ**	31.289	19	45	6.346	47	31.247	21	48	8.159	17
**MAIA NO**	13.064	4	20	3.260	47	14.353	11	18	2.090	17
**MAIA ND**	9.426	3	15	2.660	47	8.471	4	12	2.322	17
**MAIA EA**	15.709	7.5	25	4.063	47	16.250	11.3	25	3.698	17
**MAIA AR**	17.064	8	32	5.407	47	18.824	15	29	3.877	17
**MAIA BL**	7.043	3	15	2.797	47	6.824	3	11	2.270	17
**MAIA TR**	8.660	3	15	3.497	47	9.118	5	15	2.547	17

Table S3 - Baseline characteristics per engagement in the rumination task. *Baseline characteristics per engagement in the rumination task out of N=66 completing both fMRI sessions. Abbreviations: mADM, maintenance antidepressant medication; QIDS, Quick Inventory of Depressive Symptomatology; FFMQ, Five Factor Mindfulness Questionnaire; PSS, Perceived Stress Scale; RRS, Rumination Response Scale; EQ, Experience Questionnaire; MAIA, Multidimensional Assessment of Interoceptive Awareness; NO, noticing; ND, not distracting; EA, emotional awareness; AR, attention regulation; BL, body listening; TR, trusting. Values are presented as mean (x), percentage (x%) or amount (x/y). Asterisks represent significant differences between groups (**, p-val<0.01).*

Category	Participating in rumination task										Avoiding rumination task									
	MBCT + TAU					TAU					MBCT + TAU					TAU				
	value	min	max	std	N	value	min	max	std	N	value	min	max	std	N	value	min	max	std	N
**Sociodemographic characteristics**																				
**Age**	40.407	18	66	13.723	27	42.952	24	66	11.647	21	51.308	39	68	9.366	13	52.600	37	70	11.971	5
**Sex (M/F)**	6/21	-	-	-	27	4/17	-	-	-	21	3/10	-	-	-	13	0/5	-	-	-	5
**Clinical characteristics**																				
**Symptomatic (QIDS > 5)**	81.481	-	-	-	27	68.421	-	-	-	19	92.308	-	-	-	13	100.000	-	-	-	5
**mADM**	81.481	-	-	-	27	80.952	-	-	-	21	100.000	-	-	-	13	80.000	-	-	-	5
**Childhood trauma**	38.727	25	83	15.185	22	39.524	26	90	13.310	21	33.545	25	58	10.280	11	52.600	25	97	26.876	5
**Outcomes**																				
**QIDS**	8.148	1	18	4.312	27	8.684	0	17	4.547	19	12.000	5	19	4.320	13	14.000	8	21	5.701	5
**FFMQ**	43.077	27	56	7.353	26	45.905	33	61	8.130	21	43.500	32	63	9.080	12	41.600	35	56	8.444	5
**PSS**	20.846	11	31	6.175	26	19.571	9	31	6.439	21	22.750	11	34	5.707	12	25.600	15	32	6.656	5
**RRS**	54.692	29	73	10.880	26	56.714	44	71	8.361	21	50.259	32	65	9.188	12	58.800	44	68	9.149	5
**EQ**	30.423	21	43	5.637	26	32.362	19	45	7.121	21	32.650	21	48	8.428	12	27.880	21	38	7.132	5
**MAIA NO**	12.500	4	18	3.063	26	13.762	7	20	3.434	21	13.750	11	18	1.815	12	15.800	13	18	2.168	5
**MAIA ND**	9.731	5	15	2.721	26	9.048	3	14	2.598	21	8.417	4	12	2.503	12	8.600	6	11	2.074	5
**MAIA EA**	15.272	8	23.8	4.053	26	16.250	11.3	25	4.108	21	15.417	11.3	18.8	2.462	12	18.250	11.3	25	5.562	5
**MAIA AR**	16.654	8	29	5.462	26	17.571	9	32	5.427	21	18.917	15	29	3.872	12	18.600	15	26	4.336	5
**MAIA BL**	6.962	3	11	2.236	26	7.143	3	15	3.425	21	6.250	4	9	1.545	12	8.200	3	11	3.271	5
**MAIA TR**	8.846	3	15	3.258	26	8.429	3	15	3.842	21	9.333	6	15	2.605	12	8.600	5	11	2.608	5

Table S4 - Baseline characteristics per treatment group and engagement in the rumination task. *Baseline characteristics per treatment group and per engagement in the rumination task out of N=66 completing both fMRI sessions. Abbreviations: mADM, maintenance antidepressant medication; QIDS, Quick Inventory of Depressive Symptomatology; FFMQ, Five Factor Mindfulness Questionnaire; PSS, Perceived Stress Scale; RRS, Rumination Response Scale; EQ, Experience Questionnaire; MAIA, Multidimensional Assessment of Interoceptive Awareness; NO, noticing; ND, not distracting; EA, emotional awareness; AR, attention regulation; BL, body listening; TR, trusting. Values are presented as mean (x), percentage (x%) or amount (x/y). Asterisks represent significant differences between groups (**, p-val<0.01*

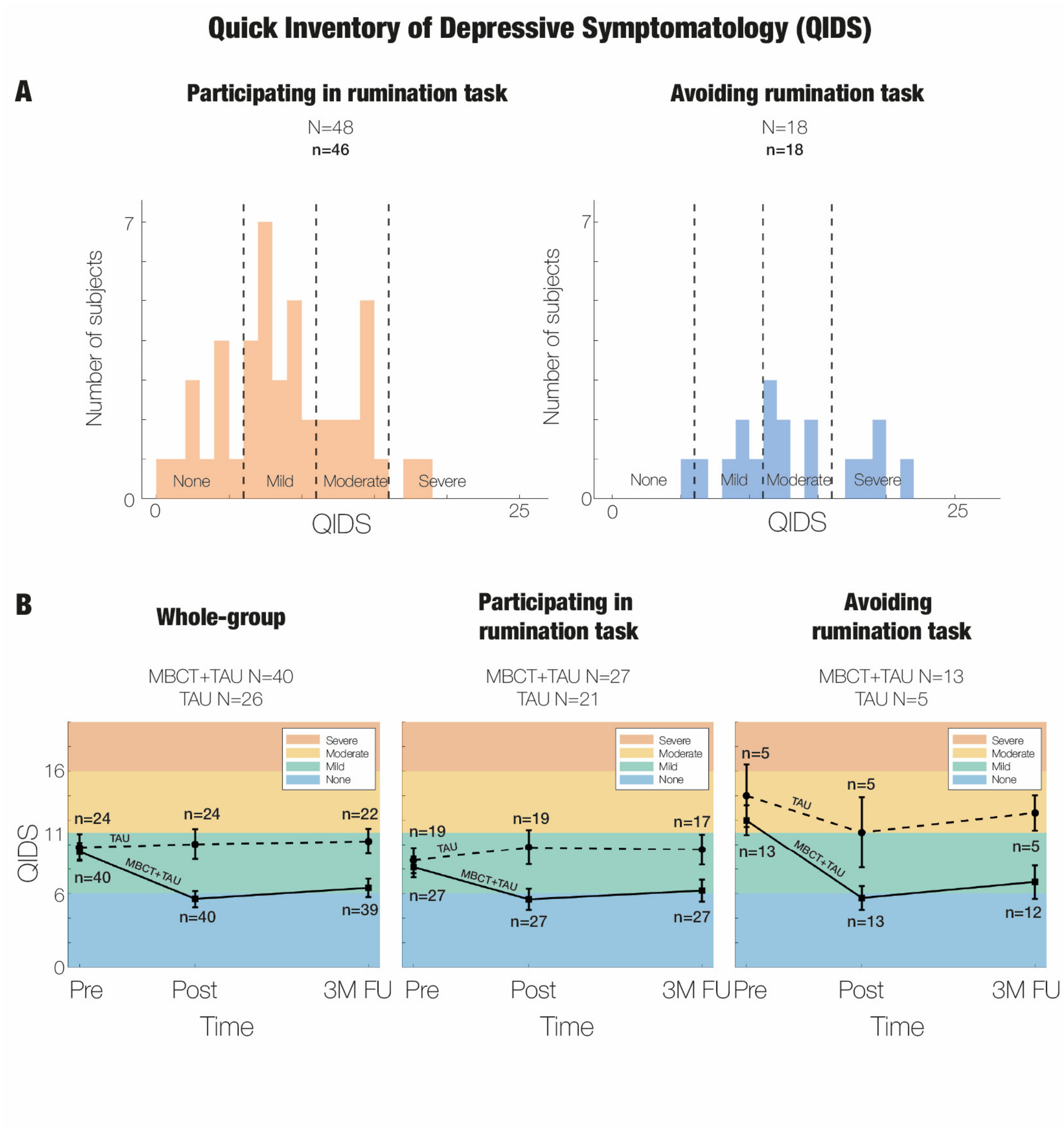

Figure S2 - Depressive symptoms across time and group

A. *Histograms of baseline depressive symptoms as measured by Quick Inventory of Depressive Symptoms (QIDS) for participants who did the rumination task (left) and participants that chose not to do the task (right).* ***B.*** *Depressive symptoms per group and timepoint across the whole group, those participating in the rumination task and those who did not. Blue represents QIDS<6 (below symptomatic threshold), green represents QIDS=6-10 (mild depressive symptoms), orange represents QIDS=11-15 (moderate depressive symptoms), and red represents QIDS>15 (severe depressive symptoms). The lines (full for MBCT+TAU and dashed for TAU) and error bars represent the mean and standard error of the mean, respectively. We detail the total number of participants with available fMRI data (N) and the participants included in this clinical analysis (n).*

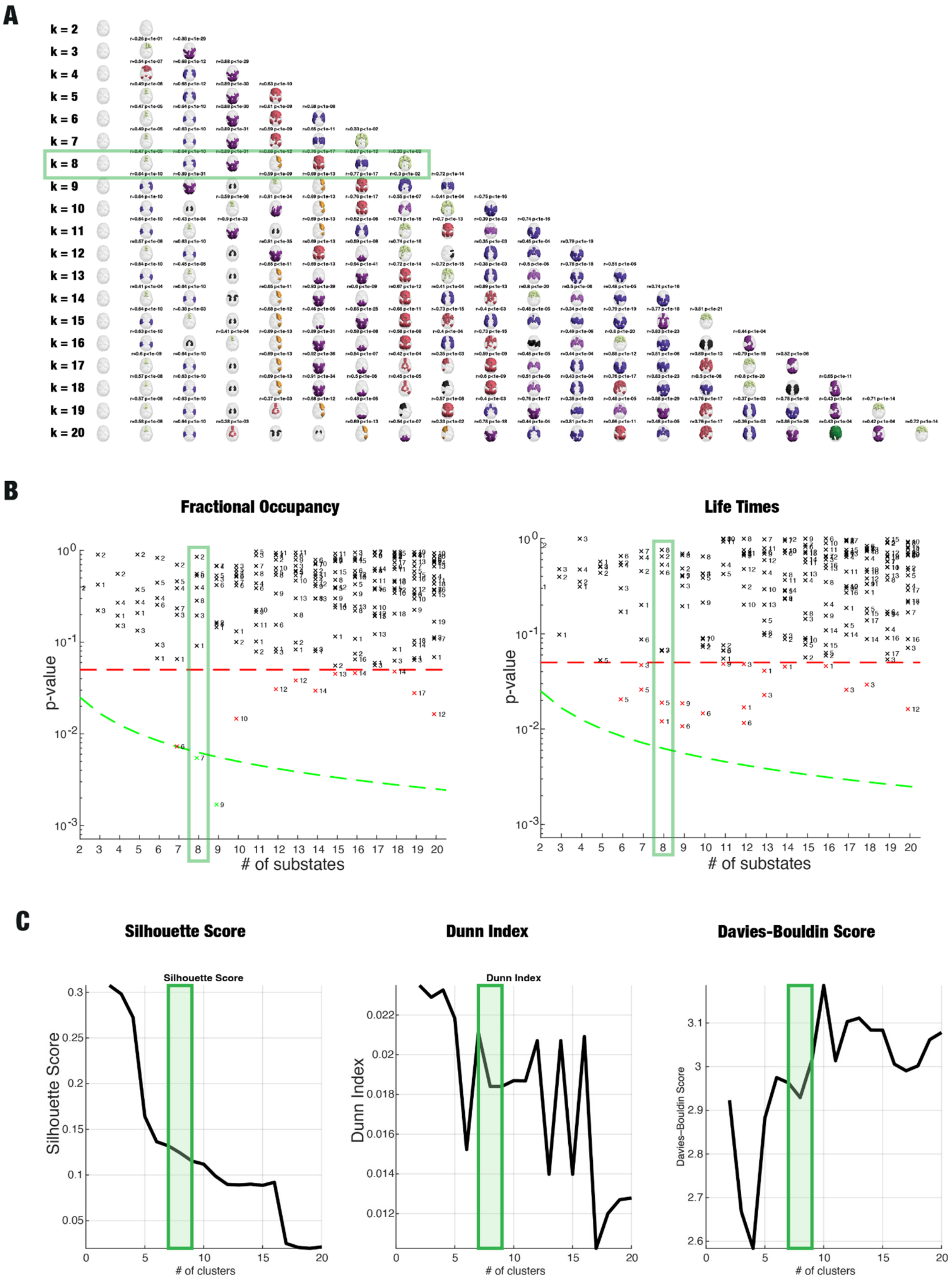

Figure S3: Identification of optimal clustering solution by the cluster quality of the data and statistically significant separation of the groups

A: Pyramidal view of the clustering solutions: *Varying number of substates with increasing number solutions of the clustering algorithm. From k=2 to k=20. The substates are represented in cortical space for the AAL parcellation. The most significant resting-network has been associated with each substate. We report the*

most significant resting-state network only when it survives the Bonferroni threshold p < 0.05/K where K is the number of substates.

B: Statistical significance of PL states between conditions: *For each clustering solution k, we plot the p-values associated with the statistical testing for the Fractional Occupancy and Lifetimes between all metastable substates for Post-Pre of MBCT+TAU versus Post-Pre of TAU groups. The black 'x' signs indicate non-significant results i.e. p-values>0.05. The red line indicates significant results (p-values<0.05), and the green line indicates significant results that were Bonferroni corrected based on the number of clustering solutions (p-values<0.05/k). As can be seen together with Figure S3A, the salience-somatomotor metastable substate is consistently significant between k=7 to k=20 (excluding k=11 and k=17) with clustering solution 8 and 9 surviving the more stringent test.*

C - Cluster evaluation across clustering solutions: *Silhouette score, Dunn index, and Davies–Bouldin score were evaluated for clustering solutions ranging from k = 2 to 20 to assess cluster quality. Higher silhouette and Dunn index values indicate better cluster separation and compactness, whereas lower Davies–Bouldin scores reflect improved clustering performance. The clustering is done in a data-driven and agnostic way to the groups and conditions ensuring the most sensible separation of the data given the cluster number "k". The choice of the clustering solution is then motivated three-fold: by the cluster quality of the data, the correlation to the functional systems, and statistically significant separation of the groups (corrected for the number of clusters analysed).*

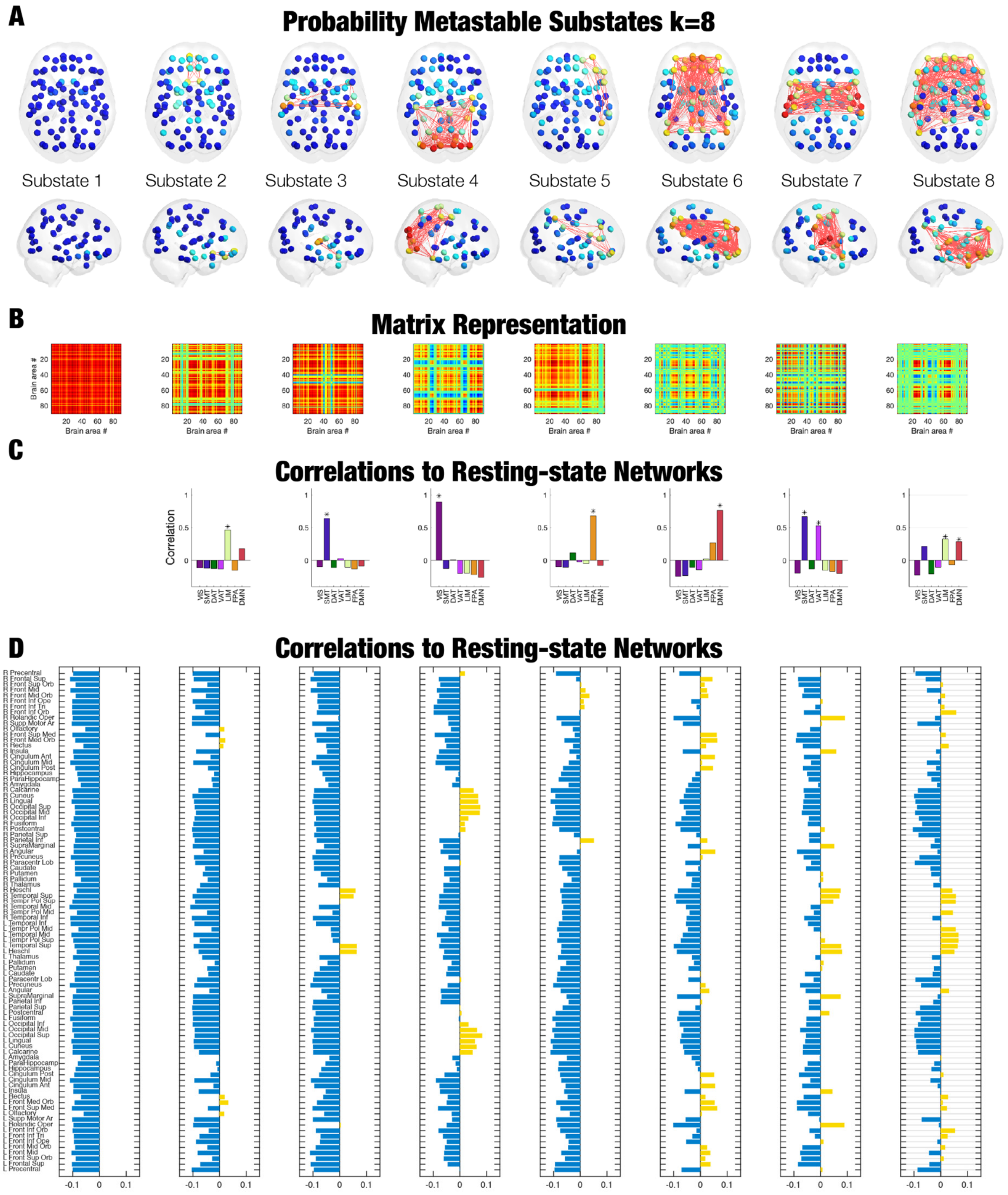

Figure S4: Phase-locking solutions for 8 substates.

A) *Cortical renders of the 8 substates. Here, we show the most important links connecting the given substates.* ***B)*** *Matrix representation of the metastable substates.* ***C)*** *Association of the metastable substates to the known 7 Resting-state networks.* ***D)*** *Regional identification for all 8 metastable substates. We chose the number of substates (8) based on the optimal clustering performance as determined by the silhouette score as well as the lowest clustering solution that resulted in Bonferroni corrected statistical significance for the Fractional Occupancy measure (see Figure S3).*

Substate	PO t-stats	PO p-val$_{perm}$	LT t-stats	LT p-val$_{perm}$
**1**	1,68	0,0504	2,78	0,0063
**2**	-0,19	0,4132	0,49	0,3749
**3**	-1,23	0,1219	-1,91	0,0326
**4**	-0,85	0,2214	-0,67	0,2766
**5**	0,59	0,294	2,29	0,0113
**6**	0,61	0,2704	0,79	0,2382
**7**	-2,87	0,0034	-1,96	0,0307
**8**	1,12	0,1513	-0,31	0,3882

Table S5. Permutation-based statistical results for the 8-PMS solution.

For each metastable substate, the table reports the t-statistic and empirical p-value from a label-permutation test for Fractional Occupancy (probability of occurrence) and Lifetime. In this non-parametric test, treatment group labels were randomly permuted across participants while preserving group sizes and keeping the learned metastable substate centroids fixed. For each of 1,000 permutations, the between-group difference in post–pre changes was recomputed, generating an empirical null distribution of the test statistic. The reported p-values correspond to the proportion of permutations yielding a statistic equal to or more extreme than the observed value. In green, we report the Bonferroni corrected p-values (<0.05/8).

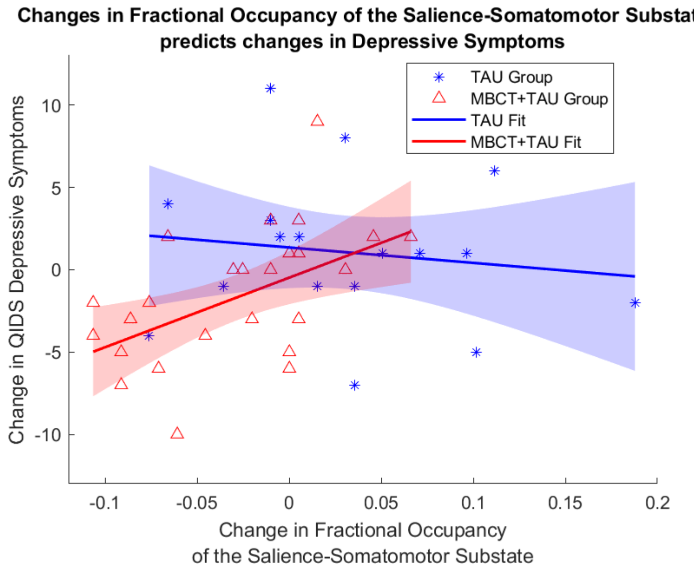

Figure S5a: Changes in the Fractional Occupancy of the Salience-somatomotor metastable substate post-treatment without outlier are associated with changes in depressive symptoms at 3 months of follow-up. *Change in the Fractional Occupancy of the Salience-somatomotor metastable substate (post-treatment - pre-treatment) in the MBCT+TAU group is significantly associated with changes in the depressive symptoms (3 months - pre-treatment), while this was not the case for the TAU group. The figure shows the linear fit of both MBCT+TAU and TAU groups with the 95% confidence intervals.*

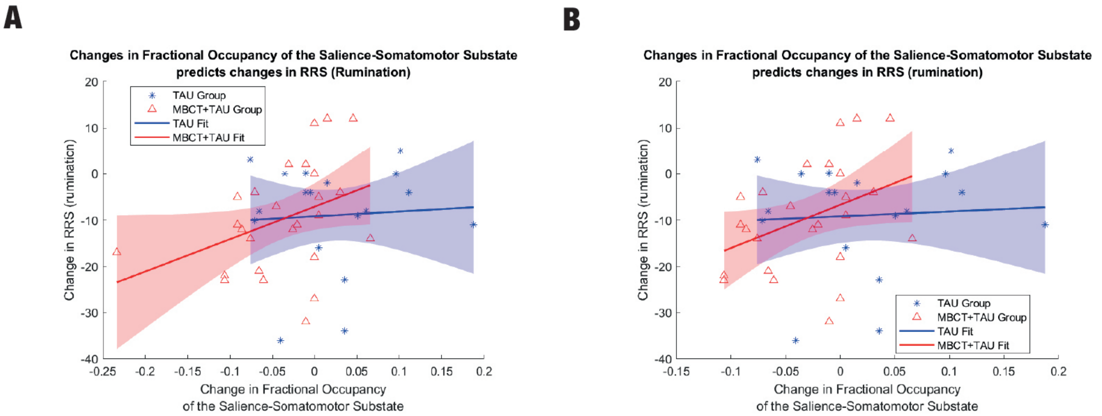

Figure S5b: Changes in the Fractional Occupancy of the Salience-somatomotor metastable substate post-treatment are associated with changes in rumination. *Change in the Fractional Occupancy of the Salience-somatomotor substate (post-treatment - pre-treatment) in the MBCT+TAU group is significantly associated with changes in rumination with (**A**) and without (**B**) outlier, while this was not the case for the TAU group. The figure shows the linear fit of both MBCT+TAU and TAU groups with the 95% confidence intervals.*

FO7 - MBCT+TAU	Pearson		Partial Pearson		Spearman		Partial Spearman		N
	Rho	P-val	Rho	P-val	Rho	P-val	Rho	P-val	
**Mental health outcomes**									
**QIDS POST**	**0.428**	**0.026**	0.386	0.069	0.339	0.084	0.350	0.101	27
**QIDS 3M**	**0.573**	**0.002**	**0.559**	**0.006**	**0.554**	**0.003**	**0.564**	**0.005**	27
**PSS**	0.272	0.179	0.231	0.301	0.124	0.546	0.109	0.629	26
**Mechanism outcomes**									
**FFMQ_TOTAL**	-0.081	0.693	-0.031	0.892	-0.061	0.766	-0.045	0.842	26
**FFMQ_observing**	-0.094	0.648	-0.004	0.986	0.049	0.812	0.117	0.604	26
**FFMQ_describe**	0.114	0.579	0.153	0.497	0.094	0.648	0.122	0.589	26
**FFMQ_ActWithAwareness**	-0.255	0.209	-0.223	0.318	-0.103	0.616	-0.077	0.734	26
**FFMQ_NonJudging**	-0.131	0.523	-0.124	0.582	-0.113	0.583	-0.129	0.569	26
**FFMQ_NonReacting**	0.134	0.514	0.152	0.499	0.094	0.650	0.080	0.723	26
**RRS_Total**	0.370	0.063	0.379	0.082	**0.410**	**0.037**	**0.426**	**0.048**	26
**RRS_Reflection**	0.259	0.201	0.215	0.336	0.216	0.289	0.207	0.355	26
**RRS_Brooding**	0.260	0.200	0.316	0.152	**0.419**	**0.033**	**0.452**	**0.035**	26
**RRS_Depression**	0.339	0.090	0.345	0.115	**0.420**	**0.033**	**0.427**	**0.048**	26
**EQ**	-0.223	0.274	-0.276	0.214	-0.233	0.252	-0.314	0.155	26
**MAIA_NO**	0.301	0.135	0.298	0.178	0.163	0.426	0.145	0.519	26
**MAIA_ND**	0.265	0.190	0.314	0.155	0.276	0.172	0.282	0.203	26
**MAIA_EA**	0.270	0.182	0.311	0.159	0.175	0.391	0.184	0.411	26
**MAIA_AR**	0.014	0.947	-0.001	0.997	-0.059	0.775	-0.088	0.696	26
**MAIA_BL**	-0.090	0.661	-0.057	0.800	-0.094	0.649	-0.098	0.663	26
**MAIA_TR**	0.000	1.000	-0.049	0.829	-0.004	0.984	-0.051	0.822	26
**Treatment engagement**									
**Attendance**	**-0.468**	**0.014**	**-0.559**	**0.006**	**-0.442**	**0.021**	**-0.495**	**0.016**	27
**Practice**	**-0.466**	**0.014**	**-0.491**	**0.017**	**-0.407**	**0.035**	**-0.424**	**0.044**	27

Table S6a: Correlations between change (post-pre) scores in Fractional Occupancy of metastable substate 7 with change (post-pre) in psychological processes and clinical outcomes for MBCT+TAU.
We report both Pearson and Spearman statistics as Pearson retains more power when the underlying assumption is linear, but relies on the assumption of linearity, whereas Spearman detects monotonic non-linear relationships. Columns: Pearson, Partial Pearson corrected for age, sex, mADM and baseline QIDS depression score, Spearman, Partial Spearman corrected for age, sex, mADM and baseline QIDS depression score, and N are the subjects that have response in all variables involved. Significant correlations are in bold and green. Rows abbreviations: QIDS post treatment and 3 months follow up; FFMQ, Five Factor Mindfulness Questionnaire, total and subscores of observing, describe, act with awareness, non-judging and non-reacting; PSS perceived stress; RRS rumination response scale, total and subscores of reflection, brooding and depression; EQ, Experience Questionnaire; MAIA, Multidimensional Assessment of Interoceptive Awareness subscores of ND, not-distracting, NO, noticing, AR, attention regulation, BL, body listening, EA, emotional awareness and TR, trusting; attendance and practice to the treatment as number of sessions (from a total of 8) and practice days per week, respectively.

FO7 - MBCT+TAU (n.o.)	Pearson		Partial Pearson		Spearman		Partial Spearman		N
	Rho	P-val	Rho	P-val	Rho	P-val	Rho	P-val	
**Mental health outcomes**									
**QIDS POST**	0.303	0.133	0.307	0.165	0.270	0.182	0.301	0.174	26
**QIDS 3M**	**0.497**	**0.010**	**0.521**	**0.013**	**0.507**	**0.008**	**0.527**	**0.012**	26
**PSS**	0.019	0.930	-0.010	0.967	0.014	0.946	0.002	0.995	25
**Mechanism outcomes**									
**FFMQ_TOTAL**	0.054	0.799	0.044	0.849	0.014	0.946	0.005	0.982	25
**FFMQ_observing**	0.224	0.282	0.280	0.220	0.175	0.404	0.227	0.323	25
**FFMQ_describe**	-0.037	0.861	-0.012	0.959	0.018	0.931	0.048	0.835	25
**FFMQ_ActWithAwareness**	0.032	0.880	0.015	0.948	0.015	0.945	0.021	0.928	25
**FFMQ_NonJudging**	-0.151	0.471	-0.191	0.407	-0.100	0.634	-0.133	0.566	25
**FFMQ_NonReacting**	0.156	0.455	0.129	0.577	0.088	0.675	0.056	0.811	25
**RRS_Total**	0.380	0.061	0.406	0.068	0.390	0.054	0.413	0.063	25
**RRS_Reflection**	0.129	0.538	0.105	0.651	0.130	0.535	0.134	0.562	25
**RRS_Brooding**	0.297	0.149	0.361	0.108	**0.420**	**0.037**	**0.455**	**0.038**	25
**RRS_Depression**	0.377	0.064	0.398	0.074	**0.430**	**0.032**	**0.445**	**0.043**	25
**EQ**	-0.003	0.989	-0.098	0.672	-0.156	0.457	-0.250	0.275	25
**MAIA_NO**	0.183	0.382	0.102	0.660	0.070	0.738	0.007	0.976	25
**MAIA_ND**	0.258	0.213	0.301	0.185	0.259	0.211	0.276	0.225	25
**MAIA_EA**	0.198	0.342	0.149	0.520	0.130	0.537	0.091	0.694	25
**MAIA_AR**	-0.020	0.925	-0.070	0.763	-0.075	0.722	-0.121	0.602	25
**MAIA_BL**	0.101	0.631	0.072	0.755	0.003	0.990	-0.027	0.908	25
**MAIA_TR**	0.131	0.532	0.063	0.787	0.076	0.717	0.026	0.913	25
**Treatment engagement**									
**Attendance**	**-0.464**	**0.017**	**-0.528**	**0.012**	**-0.407**	**0.039**	**-0.449**	**0.036**	26
**Practice**	**-0.398**	**0.044**	-0.422	0.051	-0.355	0.075	-0.369	0.091	26

Table S6b: change in FO7 without outliers and change in behavioural scores. *Columns: Pearson, Partial Pearson corrected for age, sex, mADM and baseline QIDS depression score, Spearman, Partial Spearman corrected for age, sex, mADM and baseline QIDS depression score, and N are the subjects that have response in all variables involved. Significant correlations are in bold and green. Rows abbreviations: QIDS post treatment and 3 months follow up; FFMQ, Five Factor Mindfulness Questionnaire, total and subscores of observing, describe, act with awareness, non-judging and non-reacting; PSS perceived stress; RRS rumination response scale, total and subscores of reflection, brooding and depression; EQ, Experience Questionnaire; MAIA, Multidimensional Assessment of Interoceptive Awareness subscores of ND, not-distracting, NO, noticing, AR, attention regulation, BL, body listening, EA, emotional awareness and TR, trusting; attendance and practice to the treatment as number of sessions (from a total of 8) and practice days per week, respectively.*

LT1 - MBCT+TAU	Pearson		Partial Pearson		Spearman		Partial Spearman		N
	Rho	P-val	Rho	P-val	Rho	P-val	Rho	P-val	
**Mental health outcomes**									
**QIDS POST**	-0.329	0.094	-0.231	0.290	**-0.409**	**0.034**	-0.336	0.117	27
**QIDS 3M**	**-0.415**	**0.031**	-0.349	0.102	-0.381	0.050	-0.318	0.139	27
**PSS**	**-0.400**	**0.043**	-0.395	0.069	-0.297	0.141	-0.304	0.170	26
**Mechanism outcomes**									
**FFMQ_TOTAL**	0.343	0.086	0.255	0.251	0.369	0.063	0.308	0.164	26
**FFMQ_observing**	0.148	0.471	0.093	0.682	0.122	0.552	0.080	0.724	26
**FFMQ_describe**	0.331	0.099	0.292	0.187	0.279	0.168	0.288	0.194	26
**FFMQ_ActWithAwareness**	0.123	0.551	0.074	0.743	0.114	0.578	0.058	0.796	26
**FFMQ_NonJudging**	0.352	0.078	0.291	0.189	0.240	0.237	0.198	0.378	26
**FFMQ_NonReacting**	0.239	0.240	0.125	0.578	0.260	0.199	0.175	0.435	26
**RRS_Total**	-0.367	0.065	-0.351	0.109	-0.289	0.152	-0.280	0.206	26
**RRS_Reflection**	-0.332	0.097	-0.262	0.240	**-0.396**	**0.045**	-0.321	0.145	26
**RRS_Brooding**	-0.228	0.263	-0.175	0.435	-0.168	0.411	-0.117	0.603	26
**RRS_Depression**	-0.326	0.104	-0.356	0.104	-0.222	0.275	-0.242	0.279	26
**EQ**	0.333	0.097	0.275	0.216	**0.407**	**0.039**	0.345	0.116	26
**MAIA_NO**	-0.179	0.383	-0.265	0.234	-0.203	0.320	-0.282	0.203	26
**MAIA_ND**	0.005	0.982	-0.010	0.963	0.007	0.971	0.034	0.880	26
**MAIA_EA**	0.121	0.556	0.046	0.840	0.154	0.453	0.080	0.722	26
**MAIA_AR**	0.158	0.439	0.076	0.736	0.156	0.446	0.090	0.691	26
**MAIA_BL**	0.165	0.420	0.058	0.798	0.202	0.323	0.134	0.552	26
**MAIA_TR**	0.055	0.791	0.026	0.908	0.064	0.757	0.035	0.877	26
**Treatment engagement**									
**Attendance**	0.153	0.445	0.268	0.217	0.236	0.236	0.336	0.117	27
**Practice**	**0.442**	**0.021**	**0.486**	**0.019**	**0.509**	**0.007**	**0.562**	**0.005**	27

Table S6c: change in Lifetimes 1 and change in clinical and behavioral scores. *Columns: Pearson, Partial Pearson corrected for age, sex, mADM and baseline QIDS depression score, Spearman, Partial Spearman corrected for age, sex, mADM and baseline QIDS depression score, and N are the subjects that have response in all variables involved. Significant correlations are in bold and green. Rows abbreviations: QIDS post treatment and 3 months follow up; FFMQ, Five Factor Mindfulness Questionnaire, total and subscores of observing, describe, act with awareness, non judging and non reacting; PSS perceived stress; RRS rumination response scale, total and subscores of reflection, brooding and depression; EQ, Experience Questionnaire; MAIA, Multidimensional Assessment of Interoceptive Awareness subscores of ND, not-distracting, NO, noticing, AR, attention regulation, BL, body listening, EA, emotional awareness and TR, trusting; attendance and practice to the treatment as number of sessions (from a total of 8) and practice days per week, respectively.*

LT5 - MBCT+TAU	Pearson		Partial Pearson		Spearman		Partial Spearman		N
	Rho	P-val	Rho	P-val	Rho	P-val	Rho	P-val	
**Mental health outcomes**									
**QIDS POST**	-0.064	0.750	0.066	0.764	0.027	0.895	0.158	0.470	27
**QIDS 3M**	0.040	0.845	0.115	0.600	0.084	0.678	0.161	0.464	27
**PSS**	-0.210	0.304	-0.222	0.322	-0.100	0.628	-0.116	0.608	26
**Mechanism outcomes**									
**FFMQ_TOTAL**	0.054	0.793	0.040	0.858	-0.026	0.900	-0.048	0.833	26
**FFMQ_observing**	0.357	0.073	0.284	0.201	0.238	0.242	0.146	0.518	26
**FFMQ_describe**	-0.006	0.977	-0.026	0.907	0.107	0.603	0.065	0.775	26
**FFMQ_ActWithAwareness**	0.217	0.287	0.218	0.330	0.136	0.507	0.124	0.582	26
**FFMQ_NonJudging**	-0.173	0.398	-0.152	0.501	-0.188	0.357	-0.178	0.429	26
**FFMQ_NonReacting**	-0.164	0.423	-0.133	0.554	-0.151	0.462	-0.129	0.567	26
**RRS_Total**	-0.301	0.135	-0.337	0.126	-0.158	0.441	-0.190	0.396	26
**RRS_Reflection**	**-0.417**	**0.034**	**-0.459**	**0.032**	-0.266	0.189	-0.286	0.197	26
**RRS_Brooding**	-0.125	0.542	-0.225	0.315	-0.030	0.886	-0.083	0.715	26
**RRS_Depression**	-0.250	0.219	-0.249	0.264	-0.144	0.484	-0.152	0.500	26
**EQ**	0.286	0.156	0.370	0.090	0.172	0.401	0.265	0.234	26
**MAIA_NO**	0.110	0.593	0.150	0.504	-0.009	0.964	-0.011	0.960	26
**MAIA_ND**	-0.198	0.331	-0.240	0.282	-0.089	0.667	-0.070	0.756	26
**MAIA_EA**	-0.105	0.608	-0.098	0.664	-0.131	0.523	-0.158	0.483	26
**MAIA_AR**	-0.110	0.593	-0.082	0.717	-0.110	0.593	-0.110	0.625	26
**MAIA_BL**	0.260	0.199	0.259	0.245	0.234	0.250	0.225	0.315	26
**MAIA_TR**	0.145	0.481	0.224	0.315	0.158	0.442	0.247	0.268	26
**Treatment engagement**									
**Attendance**	-0.080	0.691	-0.061	0.781	-0.146	0.467	-0.099	0.654	27
**Practice**	0.102	0.613	0.132	0.549	0.159	0.428	0.196	0.370	27

Table S6d: change in Lifetimes 5 and change in clinical and behavioral scores. *Columns: Pearson, Partial Pearson corrected for age, sex, mADM and baseline QIDS depression score, Spearman, Partial Spearman corrected for age, sex, mADM and baseline QIDS depression score, and N are the subjects that have response in all variables involved. Significant correlations are in bold and green. Rows abbreviations: QIDS post treatment and 3 months follow up; FFMQ, Five Factor Mindfulness Questionnaire, total and subscores of observing, describe, act with awareness, non judging and non reacting; PSS perceived stress; RRS rumination response scale, total and subscores of reflection, brooding and depression; EQ, Experience Questionnaire; MAIA, Multidimensional Assessment of Interoceptive Awareness subscores of ND, not-distracting, NO, noticing, AR, attention regulation, BL, body listening, EA, emotional awareness and TR, trusting; attendance and practice to the treatment as number of sessions (from a total of 8) and practice days per week, respectively.*

Theme of session	Mindfulness exercises for home practice
Session 1: Awareness and Automatic Pilot	Raisin Exercise<br>Body Scan
Session 2: Living in Our Heads	10-Minute Sitting Meditation — Mindfulness of the breath
Session 3: Gathering the Scattered Mind	Stretch and Breath Meditation<br>Mindful Movement — Formal Practice<br>3-Minute Breathing Space — Regular Version
Session 4: Recognizing Aversion	Sitting Meditation<br>Mindful Walking<br>3-Minute Breathing Space —Response Version
Session 5: Allowing/Letting Be	Working with Difficulty Meditation
Session 6: Thoughts are Not Facts	10-Minute Sitting Meditation<br>20-Minute Sitting Meditation<br>Bells at 5 Minutes, 10 Minutes, 15 Minutes, 20 Minutes, and 30 Minutes
Session 7: "How Can I Best Take Care of Myself?"	Adapted breathing space - action step
Session 8: Maintaining and Extending New Learning	

Table S7: Brief overview of MBCT practices

Overview of an 8-week MBCT course. In the first half of the program (sessions 1-4) the basics of mindfulness are taught. Participants are taught to become increasingly aware of changes in their attention including mind wandering in daily life. They learn to note change and flux in thoughts, feelings, and physical sensations without judgment, and having noticed that the mind is wandering, they learn to bring it back to present moment body sensations. In addition, participants are taught to become aware of the relationship between negative thoughts and affective dynamics. Practices vary from brief 3 minutes practices to pause and check-in during daily life (the breathing space) to longer 20-40 minutes guided practices. In the second half of the program (sessions 5-8) becoming aware of and dealing with difficulty and negative thoughts (e.g. rumination) and emotions is taught. In session 5, practices focus on dealing with difficult emotions and thoughts by noting the body part affected, and by bringing awareness to it, opening and softening the sensation with the breathing rather than being tightened around it. In session 6, practices focus on how low mood can cause negative thoughts to arise, which are in turn taken to be true, the adhesiveness of the thoughts, and how they may be held and seen in awareness as mental events (decentering or meta-cognitive awareness). In session 7 and 8 participants are taught to become more aware of their own unique warning signs of impending depression and develop specific preventative action plans. Adapted from Segal ZV, Williams JM, Teasdale JD. Mindfulness-based cognitive therapy for depression: a new approach to preventing relapse. New York: Guilford Press; 2002 (13).

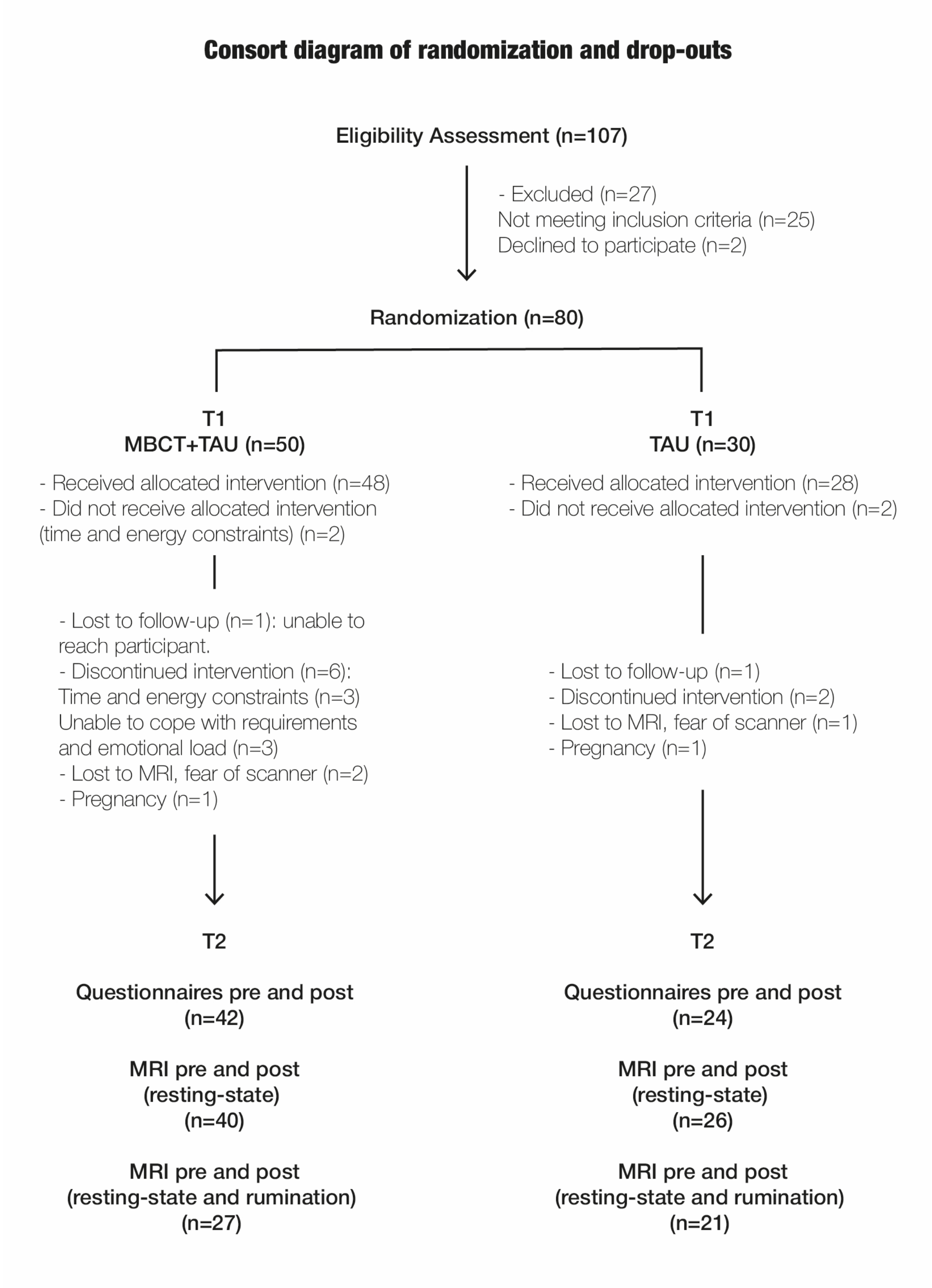

S6: Consort diagram of randomization and dropouts - adapted from van der Velden et al., 2023 (14).

Supplementary References:

1. Crane RS, Kuyken W (2019): The Mindfulness-Based Interventions: Teaching Assessment Criteria (MBI:TAC): reflections on implementation and development. *Curr Opin Psychol* 28: 6–10.
2. Crane RS, Eames C, Kuyken W, Hastings RP, Williams JMG, Bartley T, *et al.* (2013): Development and validation of the mindfulness-based interventions - teaching assessment criteria (MBI:TAC). *Assessment* 20: 681–688.
3. Karl A, Williams MJ, Cardy J, Kuyken W, Crane C (2018): Dispositional self-compassion and responses to mood challenge in people at risk for depressive relapse/recurrence. *Clinical Psychology & Psychotherapy* 25: 621–633.
4. Glerean E, Salmi J, Lahnakoski JM, Jääskeläinen IP, Sams M (2012): Functional Magnetic Resonance Imaging Phase Synchronization as a Measure of Dynamic Functional Connectivity. *Brain Connectivity*, vol. 2. pp 91–101.
5. Cabral J, Vidaurre D, Marques P, Magalhães R, Silva Moreira P, Miguel Soares J, *et al.* (2017): Cognitive performance in healthy older adults relates to spontaneous switching between states of functional connectivity during rest. *Sci Rep* 7: 5135.
6. Vohryzek J, Deco G, Cessac B, Kringelbach ML, Cabral J (2020): Ghost Attractors in Spontaneous Brain Activity: Recurrent Excursions Into Functionally-Relevant BOLD Phase-Locking States. *Front Syst Neurosci* 14: 20.
7. Lord L-D, Expert P, Atasoy S, Roseman L, Rapuano K, Lambiotte R, *et al.* (2019): Dynamical exploration of the repertoire of brain networks at rest is modulated by psilocybin. *Neuroimage* 199: 127–142.
8. Cahart M-S, Dell'Acqua F, Giampietro V, Cabral J, Timmers M, Streffer J, *et al.* (2022): Test-retest reliability of time-varying patterns of brain activity across single band and multiband resting-state functional magnetic resonance imaging in healthy older adults. *Front Hum Neurosci* 16: 980280.
9. Cahart M-S, Giampietro V, O'Daly O (2025): Atypical attentional network dynamics in adolescent depression during emotional movie viewing. *Soc Cogn Affect Neurosci* 20. https://doi.org/10.1093/scan/nsaf011
10. Cahart M-S, Giampietro V, Naysmith L, Muraz M, Zelaya F, Williams SCR, O'Daly O (2024):

Anhedonia severity mediates the relationship between attentional networks recruitment and emotional blunting during music listening. *Scientific Reports* 14: 20040.

11. Alonso Martínez S, Deco G, Ter Horst GJ, Cabral J (2020): The Dynamics of Functional Brain Networks Associated With Depressive Symptoms in a Nonclinical Sample. *Front Neural Circuits* 14: 570583.
12. Thomas Yeo BT, Krienen FM, Sepulcre J, Sabuncu MR, Lashkari D, Hollinshead M, *et al.* (2011): The organization of the human cerebral cortex estimated by intrinsic functional connectivity. *J Neurophysiol*. https://doi.org/10.1152/jn.00338.2011
13. Segal ZV, Williams JMG, Teasdale JD (2012): *Mindfulness-Based Cognitive Therapy for Depression*. Guilford Press.
14. van der Velden AM, Scholl J, Elmholdt E-M, Fjorback LO, Harmer CJ, Lazar SW, *et al.* (2023): Mindfulness Training Changes Brain Dynamics During Depressive Rumination: A Randomized Controlled Trial. *Biol Psychiatry* 93: 233–242.
